# Hematological and blood chemistry parameters of a *Podocnemis vogli* and *P. unifilis* captive population in Colombia

**DOI:** 10.3389/fvets.2022.961609

**Published:** 2022-09-15

**Authors:** Cristian Rodríguez-Almonacid, Gustavo Fuentes-Rodríguez, Leidy P. González, Carlos Moreno-Torres, Nubia E. Matta

**Affiliations:** ^1^Grupo Caracterización Genética e Inmunología, Departamento de Biología, Facultad de Ciencias, Universidad Nacional de Colombia, Bogotá, Colombia; ^2^Departamento de Salud Animal, Facultad de Medicina Veterinaria y de Zootecnia, Universidad Nacional de Colombia, Bogotá, Colombia

**Keywords:** biochemical parameters, hematology, neotropic, Podocnemididae, reference interval values, serum, turtles

## Abstract

The Podocnemididae family is seriously affected by anthropogenic factors, which is why almost all of their family members are threatened, according to the IUCN red list. The biology and ecology of these species, as well as the hematological and serum chemistry reference intervals that allow clinical action and decision-making conservation programs, are poorly known. Based on this, the objective of this study was to establish the hematological and blood chemistry parameters of the Savannah side-necked turtle (*Podocnemis vogli*) and Yellow-spotted river turtle (*Podocnemis unifilis*) maintained in captivity at the Estación de Biología Tropical Roberto Franco (Villavicencio-Colombia). Forty-nine captive turtles of the species *P. vogli* (n = 28) and *P. unifilis* (n = 21) were sampled to determine hematological and serum chemistry parameters. Blood samples were taken from the jugular veins of both male and female turtles across both species. Student's *t*-test and Mann–Whitney–Wilcoxon tests were used to compare values between the parameters evaluated against genders and sizes. Reference intervals were calculated for the hematological and biochemical values of each species. Some assessed parameters demonstrated significant differences between the males and females of both species. Most of the analyzed parameters exhibited similar reference intervals in both species. In this study, we report values and propose the hematological and serum chemistry reference intervals for *P. vogli* and *P. unifilis*, which can be used in the clinical diagnosis of these reptiles and in future research.

## Introduction

The Savannah side-necked turtle (*Podocnemis vogli*) and Yellow-spotted river turtle (*P. unifilis*) are two of eight species of the Podocnemididae family distributed in the north of South America that are seriously affected by anthropogenic factors, as they are a source of meat, eggs, oil, and other means for local inhabitants, leading to their overexploitation (1). Thus, according to the IUCN Red List, five out of the six species of the *Podocnemis* genera are threatened, where *P. unifilis* is listed as vulnerable (VU) (2), while *P. vogli* is classified as least concern (LC) according to the Red Book of Reptiles of Colombia, although it is beginning to be extracted from nature due to the decrease in other podocnemidids traditionally consumed (3). Threats like illegal trafficking have caused these tortoises to be recurrently seized by authorities, leaving them in the care of rescue centers that work to restore their health and promote their conservation. To assess the health status of chelonians, physical examinations and evaluation of hematological and blood chemistry parameters are necessary, which must be compared with reference intervals of healthy specimens to identify individuals who require care and intervention; however, reference information of *P. unifilis* about hematology and blood chemistry is limited, while it is nonexistent for *P. vogli*.

The data about podocnemidids' hematology and blood chemistry available are restricted to reports of the captive populations of *Peltocephalus dumerilianus, Podocnemis expansa*, and *P. unifilis* (4–8) and of the wild populations of *Podocnemis expansa*, and *Podocnemis erythrocephala* (9, 10). Despite being scarce, the available research is of great importance, as it provides fundamental information to evaluate the state of health, prognosis, diagnosis, the effectiveness of rehabilitation protocols, and the variation of blood analytes in different intrinsic (age, sex, species) and extrinsic conditions (season, temperature, diet, diseases, etc.) of *in-situ* and *ex-situ* turtles (11, 12).

Based on that, this study aimed to assess the hematological and blood chemistry parameters of *Podocnemis vogli* and *P. unifilis* maintained in captivity and to propose reference intervals that lead to the interpretation of these parameters in the medical assessment of both species.

## Materials and methods

### Population

Forty-nine turtles belonged to *Podocnemis vogli* (*n* = 28; 17 females, 11 males) and *P. unifilis* (*n* = 21; 15 females, six males) species were sampled. Sampling was carried out in September 2019 at Estación de Biología Tropical Roberto Franco of the Universidad Nacional de Colombia, located in Villavicencio-Meta, Colombia (latitude 4.13°, longitude 73.63°, and 419 m.a.s.l). Local temperatures oscillate between 20 and 32 °C with a mean relative humidity of 76%, an average annual rainfall of 4.008 mm, and a unimodal rainfall regime.

Due to a lack of information in clinic history about the date of birth or the date of the capture of turtles sampled, age could not be established. However, data available indicate that all *P. unifilis* have lived in captivity at Estación de Biología Tropical Roberto Franco for 14 to 27 years; moreover, all *P. vogli* turtles have lived in captivity for 12–20 years; therefore, all sampled individuals were adults. Additionally, all turtles sampled in this study were apparently healthy (active, alert, without external wounds, ectoparasites, or blood parasite infection). Sex and morphometric measurements such as maximal carapace length (MCL), maximal carapace width (MCW), maximal plastron length (MPL), carapace height (CH), and weight were determined.

### Blood samples

Blood was obtained from the jugular vein using 1 mL syringes without anticoagulant and a 25 G x 1-inch needle. All samples were collected one day before feeding to avoid the influence of food on blood tests. For hematological analysis, 200 μL of blood was deposited in vials with sodium heparin (100 UI/mL, Liquemine, Roche), carefully mixed, and stored at 4°C until the analysis. Fresh blood was used to make four thin blood smears and measure hematocrit. Blood smears were dried using airflow and fixed using absolute methanol for 5 min. Blood films were stained using Wright and Giemsa, respectively, for hematological and parasitological studies. Serum was obtained using separator microtubes (Liuyang Sanli Medical Technology Development Co Ltd), centrifuged at 4,000 rpm for 10 min, and stored at−20°C until the analysis.

### Hematological analyses

Hematocrit was determined through microhematocrit centrifugation at 12,000 *g* for 5 min (13). Hemoglobin concentration was measured by spectrophometry using BioSystems BTS-350 (BioSystems, S.A., Spain). Red and white blood cell counts (RBC, WBC) were made manually using a 1:100 Natt-Herrick diluent solution in a Neubauer chamber (14). Erythrocytic indices such as mean corpuscular volume (MCV), mean corpuscular hemoglobin (MCH), and mean corpuscular hemoglobin concentration (MCHC) were calculated following the guidelines of Eatwell et al. (15). A differential count was carried out on Wright stained blood smears, counting 100 leukocytes on a 100x objective lens and recorded as a percentage (15). Giemsa-stained blood smears were examined for intracellular and extracellular haemoparasites using an Olympus BX43 microscope (Olympus Corp.; Tokyo, Japan). Digital images of blood cells were obtained using an Olympus DP27 digital camera and processed with the cellSens software (v. 1.13; Olympus Corp.; Tokio, Japan).

### Blood chemistry analyses

Total solids (TS) were measured using a hand refractometer (Scientific^®^, China). Serum biochemistry analytes were measured by spectrophometry using BioSystems BTS-350 (BioSystems, S.A., Spain) and Spinreact kits (Spinreact, Spain). Analytes measured included glucose, albumin, aspartate aminotransferase (AST), alanine aminotransferase (ALT), alkaline phosphatase (ALP), cholesterol, uric acid, and creatinine. Non-chemical analyses were made for total blood.

### Statistical analyses

Descriptive statistics were made for all parameters. The XLStat package v.2020.1.3 (16) was used to assess the normality of data through the Shapiro–Wilk test, while the presence of outliers was determined through the Dixon test, with a 95% confidence interval. The outliers were manually removed, and the normality tests were repeated.

Student's *t*-test and the Mann–Whitney–Wilcoxon tests were performed to assess significant differences between the parameters evaluated against genders and sizes; the R software (v. 4.0.0) was used for this. The establishment of the reference intervals was carried out following the guidelines of the American Society for Veterinary Clinical Pathologists (ASVCP) (17), calculating a 95% reference interval using parametric methods for normally distributed parameters, while robust methods were used for non-parametric parameters, following the guidelines proposed by the Clinical and Laboratory Standards Institute in the EP28-A3c guide (18), using MedCalc^®^ v.19.4.0 (19). Additionally, the 90% confidence intervals of the lower and upper limits were calculated for each parameter (20).

## Results

### Samples

Forty-nine turtles of *Podocnemis vogli* (*n* = 28) and *P. unifilis (n* = 21) that live captive at Estación de Biología Tropical Roberto Franco were sampled. Morphometric data of these turtles are illustrated in Table 1. Overall, for both species, females were significantly bigger than males in all morphological parameters evaluated (*P* < 0.05); additionally, the Yellow-spotted river turtles (*P. unifilis*) were, on average, larger than the Savannah side-necked turtles (*P. vogli*).

**Table 1:** Savannah side-necked turtle (*P. vogli*) and Yellow-spotted river turtle (*P. unifilis*) morphometric parameters.

**A**	**Female (*n = * 17)**			**Male (*n = *11)**		
**Morphometric parameter**	**Mean**	**SD**	**Range**	**Mean**	**SD**	**Range**
MCL (cm)	24.2	4.7	16.1–30.5	18.2	2.4	15.1–22.4
MCW (cm)	18.2	3.4	12.3–21.9	14.1	1.4	12.3–16.7
MPL (cm)	21.7	4.2	14.2–27.0	16.3	2.6	13.2–21.8
CH (cm)	8.2	1.6	5.9–10.8	6.7	0.8	5.4–8.2
Weight (kg)	2	1	0.6–3.4	0.9	0.3	0.7–1.6
**B**	**Female (*n = * 15)**			**Male (*n = * 6)**		
**Morphometric parameter**	**Mean**	**SD**	**Range**	**Mean**	**SD**	**Range**
MCL (cm)	29.8	3.1	24.5–33.9	24.3	1.7	22.4–26.6
MCW (cm)	21.7	2.2	18.4–25.9	18.4	1.2	17.5–20.5
MPL (cm)	26.7	2.8	22–30	21.9	2	19.5–24.2
CH (cm)	10.6	1.1	8.7–12.5	9.3	0.6	8.3–10.1
Weight (kg)	3.2	0.9	1.8–4.6	1.9	0.5	1.3–2.5

MCL, maximal carapace length; MCW, maximal carapace width; MPL, maximal plastron length; CH, carapace height. (A) Savannah side-necked turtle (*Podocnemis vogli*) and (B) Yellow-spotted river turtle (*P. unifilis*) from Estación de Biología Tropical Roberto Franco. Villavicencio – Colombia. All parameters showed significant differences between the sexes (P < 0.05).

### Hematology and serum chemistry

**Figure 1:**
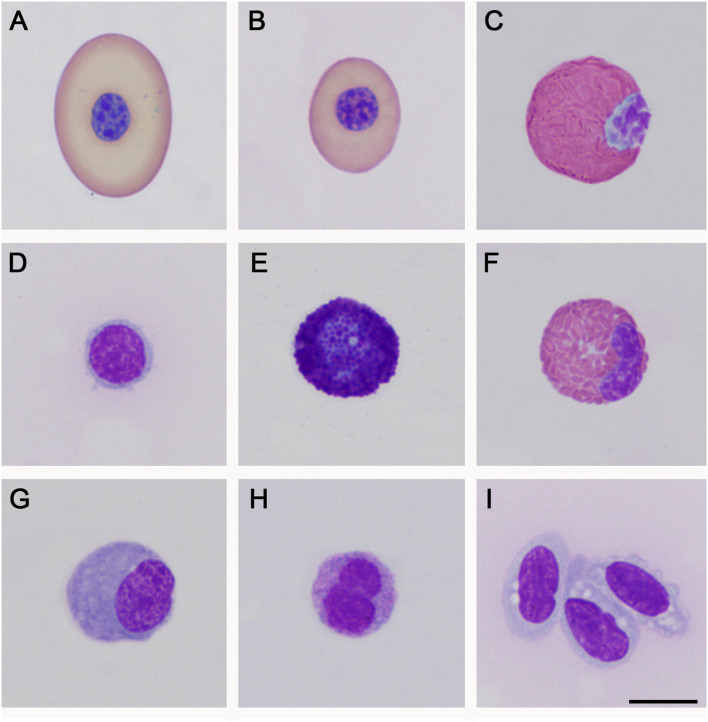
Peripheral blood cells of *Podocnemis vogli*. **(A)**: Mature erythrocyte; **(B)**: Polychromatophil; **(C)**: Heterophil; **(D)**: Immature lymphocyte; **(E)**: Basophil; **(F)**: Eosinophil; **(G)**: Monocyte; **(H)**: Azurophil-like; **(I)**: Thrombocyte. Wright staining. Bar: 10 μm.

**Figure 2:**
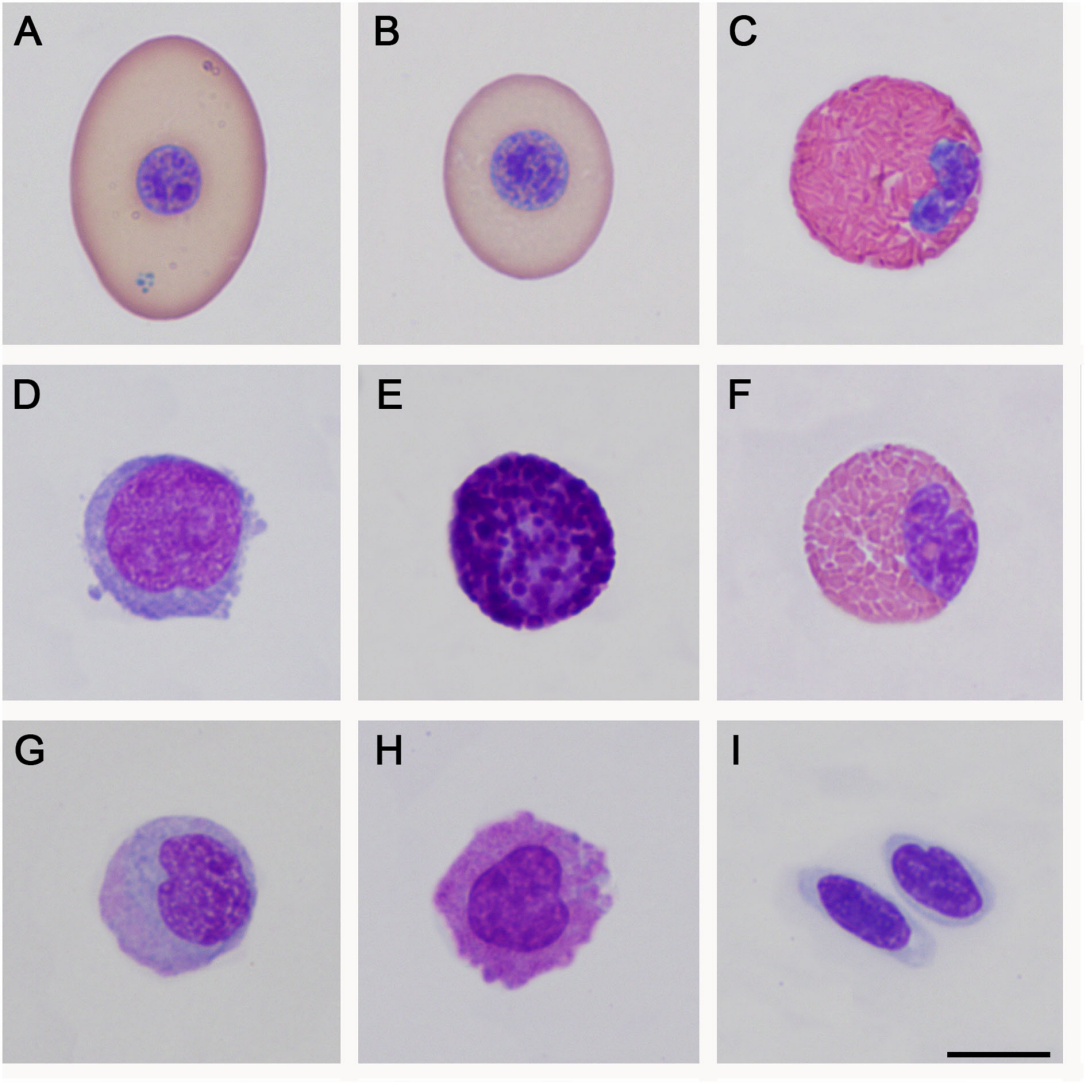
Peripheral blood cells of *Podocnemis unifilis*. **(A)**: Mature erythrocyte; **(B)**: Polycromatophil; **(C)**: Heterophil; **(D)**: Mature lymphocyte; **(E)**: Basophil; **(F)**: Eosinophil; **(G)**: Monocyte; **(H)**: Azurophil-like; **(I)**: Thrombocyte. Wright staining. Bar: 10 μm.

Morphometrical analysis of peripheral blood cells allowed us to detect that, in *P. vogli*, the average mature red blood cells (24.4 μm X 15.7 μm) are smaller than those found in *P. unifilis* (25.6 μm X 18.1 μm). Leukocytes showed similar characteristics to those reported in other turtles, such as *Chelonia mydas* and *Lepidochelys olivacea* (11, 21, 22). Blood cells found in *P. vogli* and *P. unifilis* are shown in Figures 1, 2, respectively.

The hematological and serum chemistry values of *P. vogli* are illustrated in Table 2. Mean corpuscular volume (MCV), the percentage of eosinophils, and the concentration of creatinine showed significantly statistical differences between males and females (*P* = 0.01; *P* = 0.04, and *P* = 0.01, respectively). Table 3 includes the hematological and serum chemistry values for *P. unifilis*. For this species, the percentage of monocytes and azurophils, as well as the concentration of AST, cholesterol, and creatinine, showed significantly statistical differences between both sexes (*P* = 0.01; *P* = 0.02; *P* = 0.009; *P* = 0.002 and *P* = 0.007, respectively). Parameters with significant differences between the sexes are detailed in Supplementary Table 1. After removing outliers from different parameters, the *n* was lower than 20; therefore, it is not recommended to establish reference intervals for these parameters.

**Table 2:** Hematological and blood chemical values of the Savannah side-necked turtle (*P. vogli*).

**Analytes**	***n***	**Mean**	**SD**	**Median**	**Min–Max**	**LL (90% IC)**	**UL (90% IC)**	***p-*** **value**
PCV (%)	22	24.8	2.5	24.2	21.0–30.0	19.9 (18.3–21.4)	29.6 (30.1–31.1)	0.23
TS (g/dL)	25	4.2	1.2	4.3	1.5–7.0	1.9 (1.2–2.6)	6.5 (7.7–7.1)	0.43
Hemoglobin (g/dL)	24	6.5	1.4	6.2	4.2–9.7	3.7 (2.8–4.5)	9.2 (9.3–10.0)	0.50
RBC (10^6^/μL)	28	0.23	0.07	0.23	0.12–0.37	0.09 (0.04–0.12)	0.37 (0.33–0.41)	0.20
WBC (10^3^/μL)	28	3.55	1.20	3.41	1.39–5.55	1.19 (0.5–1.8)	5.91 (5.2–6.5)	0.27
MCV (fL)^a^	22	1,089	367	1,025	635–2,000	369 (143–596)	1,810 (2,000–2,036)	0.05
MCH (pg)	24	324.1	129.0	298.6	135.1–608.3	65.8 (0–143.5)	582.3 (608.5–660.0)	0.17
MCHC (g/dL)	19	26.0	4.7	26.2	17.5–35.3	16.4 (13.1–19.6)	35.5 (35.2–38.7)	0.61
Heterophils (%)	28	45.8	12.4	43.5	24.0–73.0	21.6 (14.8–28.3)	70.0 (73.2–76.7)	0.73
Lymphocytes (%)	28	25.8	11.0	25.5	1.0–47.0	4.3 (0–10.2)	47.3 (47.3–53.2)	0.93
Eosinophils (%)^a^	28	16.9	6.2	15.5	5.0–28.0	4.7 (1.2–8.0)	29.0 (28.6–32.4)	0.30
Monocytes (%)	28	6.6	2.8	6.0	3.0–14.0	0.2 (0–1.7)	12.2 (14.7–14.1)	0.01
Basophils (%)^b^	27	4.0	3.1	4.0	0.0–10.0	-	-	0.03
Azurophils (%)^b^	27	0.3	0.6	0.0	0.0–2.0	-	-	0.00
Heterophils (10^3^/μL)	28	1.50	0.40	1.42	0.79–2.39	0.71 (0.46–0.89)	2.29 (2.00–2.42)	0.73
Lymphocytes (10^3^/μL)	28	0.84	0.36	0.84	0.03–1.54	0.14 (0–0.33)	1.55 (1.30–1.68)	0.93
Eosinophils (10^3^/μL)	28	0.55	0.20	0.51	0.16–0.92	0.15 (0.04–0.26)	0.95 (0.83–1.06)	0.30
Monocytes (10^3^/μL)	28	0.22	0.09	0.20	0.10–0.46	0.01 (0–0.05)	0.40 (0.32–0.46)	0.01
Basophils (10^3^/μL) ^b^	24	0.13	0.10	0.11	0.0–0.33	0	0.33 (0.26–0.39)	0.02
Azurophils (10^3^/μL) ^b^	28	0.01	0.02	0.00	0.0–0.07	-	-	0.00
Glucose (mg/dL)	25	68.0	14.3	67.0	47.0–99.0	39.9 (31.6–48.2)	96.2 (99.8–104.4)	0.16
Albumin (g/dL)	25	1.5	0.3	1.5	0.8–2.2	1.0 (0.78–1.12)	2.1 (2.9–2.2)	0.37
ALT (U/L)	24	4.9	1.6	5.0	1.0–8.0	1.7 (0.80–2.68)	8.0 (8.0–8.9)	0.31
AST (U/L)	24	129.1	36.5	123.0	79.0–215.0	57.6 (36.0–79.1)	200.7 (215.1–222.2)	0.10
ALP (U/L)	25	91.9	30.5	91.0	32.0–151.0	32.1 (14.5–49.7)	151.6 (151.0–169.2)	0.94
Cholesterol (mg/dL)	24	120.0	32.8	115.5	43.0–177.0	55.6 (36.2–74.9)	184.3 (177.9–203.6)	0.84
Uric Acid (mg/dL)	25	1.1	0.3	1.0	0.6–1.8	0.4 (0.19–0.56)	1.7 (1.4–1.8)	0.09
Creatinine (mg/dL) ^a.b^	25	0.3	0.1	0.3	0.2–0.4	-	-	0.00

SD, standard deviation; LL, lower limit; UL, upper limit; CI, confidence interval; TS, total solids; RBC, red blood cell count; WBC, white blood cell count; MCV, mean corpuscular volume; MHC, mean hemoglobin concentration; MCHC, mean corpuscular hemoglobin concentration; ALT, alanine aminotransferase; AST, aspartate aminotransferase; ALP, alkaline phosphatase; p-value, p-value for normality test.

a Parameters with significant differences between the sexes (P < 0.05); for specific information. see Supplementary Table 1.

b RI and CI were not established due to the inability of the software to perform the bootstrap.

**Table 3:** Hematological and blood chemical values of the Yellow-spotted river turtle (*P. unifilis*).

**Analytes**	***n***	**Mean**	**SD**	**Median**	**Min–max**	**LL (90% IC)**	**UL (90% IC)**	***p-*** **value**
PCV (%)	21	23.0	4.3	24.0	14.0–30.0	14.7 (12.0–17.3)	31.4 (28.7–34.0)	0.81
TS (g/dL)	21	4.05	0.79	4.10	2.4–5.6	2.51 (2.0–3.0)	5.60 (5.0–6.0)	0.72
Hemoglobin (g/dL)	19	6.38	1.28	6.70	4.0–8.7	3.88 (3.0–4.7)	8.89 (8.0–9.7)	0.26
RBC (10^6^/μL)	19	0.165	0.031	0.163	0.110–0.218	0.11 (0.08–0.12)	0.23 (0.20–0.24)	0.92
WBC (10^3^/μL)	20	3.33	1.11	3,413.3	1.332–5.162	1.1 (0.42–1.87)	5.5 (4.7–6.2)	0.66
MCV (fL)	19	1,413	318	1,481	643–1,935	790 (578–1,001)	2,036 (1,824–2,248)	0.33
MCH (pg)	17	388.7	77.2	363.6	276.9–580.0	237.3 (182.8–291.7)	540.0 (485.5–594.4)	0.30
MCHC (g/dL)	19	28.9	7.6	26.2	21.1–47.1	8.4 (3.7–16.6)	42.7 (34.8–50.1)	0.00
Heterophils (%)	21	54.8	10.4	52.0	34.0–74.0	34.5 (27.9–41.0)	75.1 (68.5–81.6)	0.39
Lymphocytes (%)	21	24.9	7.6	26.0	8.0–39.0	10.1 (5.3–14.8)	39.7 (34.9–44.4)	0.67
Eosinophils (%)	20	8.7	5.7	8.0	1.0–19.0	0 (0–1.2)	19.7 (16.0–23.4)	0.28
Monocytes (%)^a^.^b^	21	6.5	3.5	6.0	2.0–12.0	-	-	0.04
Basophils (%)	20	3.0	2.4	3	0–8.0	0.00	7.63 (6.0–9.1)	0.12
Azurophils (%)^a^.^b^	21	0.6	0.7	0.0	0.0–2.0	-	-	0.00
Heterophils (10^3^/μL)	20	1.89	0.84	1,807.08	0.68–3.47	0.25 (0–0.79)	3.53 (2.9–4.0)	0.21
Lymphocytes (10^3^/μL)	20	0.81	0.34	779.22	0.29–1.42	0.14 (0–0.36)	1.48 (1.2–1.7)	0.47
Eosinophils (10^3^/μL)	20	0.30	0.21	287.49	0.03–0.68	0 (0–0.03)	0.71 (0.57–0.84)	0.16
Monocytes (10^3^/μL)	20	0.21	0.14	154.85	0.07–0.47	0 (0)	0.49 (0.35–0.61)	0.00
Basophils (10^3^/μL)	16	0.08	0.07	0.07	0.00–0.25	0 (0)	0.23 (0.17–0.27)	0.12
Azurophils (10^3^/μL) ^b^	21	0.02	0.03	0.00	0.00–0.09	-	-	0.00
Glucose (mg/dL)	20	38.7	19.6	31.5	19.0–86.0	0 (0–3.09)	74.7 (56.7–93.7)	0.00
Albumin (g/dL)	19	1.5	0.2	1.5	1.1–1.9	1.14 (1.02–1.25)	1.81 (1.6–1.9)	0.18
ALT (U/L)^b^	19	5.9	4.0	5.0	1.0–13.0	-	-	0.03
AST (U/L)^a^	19	95.9	30.3	87.0	52.0–166.0	19.0 (3.01–46.1)	154.1 (125.0–184.0)	0.04
ALP (U/L)	18	95.6	30.5	93.5	60.0–167.0	35.8 (14.9–56.7)	155.3 (134.4–176.1)	0.13
Cholesterol (mg/dL)^a^	20	90.9	41.0	79.5	35.0–166.0	10.6 (0–37.1)	171.2 (144.6–197.7)	0.11
Uric Acid (mg/dL)	20	1.3	0.3	1.1	1.0–2.0	0.33 (0.17–0.65)	1.90 (1.6–2.2)	0.00
Creatinine (mg/dL)^a^.^b^	20	0.2	0.1	0.2	0.1–0.4	-	-	0.00

SD, standard deviation; LL, lower limit; UL, upper limit; CI, confidence interval; TS, total solids; RBC, red blood cell count; WBC, white blood cell count; MCV, mean corpuscular volume; MHC, mean hemoglobin concentration; MCHC, mean corpuscular hemoglobin concentration; ALT, alanine aminotransferase; AST, aspartate aminotransferase; ALP, alkaline phosphatase; p-value, p-value for normality test.

a Parameters with significant differences between the sexes (P < 0.05); for specific information. see Supplementary Table 1.

b RI and CI were not established due to the inability of the software to perform Bootstrap.

## Discussion

This study reports for the first time hematological and serum chemistry values and reference intervals for *Podocnemis vogli;* moreover, new data on the hematology of *P. unifilis* are described, and serum chemistry values for this species are published for the first time.

Sexual dimorphism has been reported previously for members of the Podocnemididae family, such as *P. erythrocephala* and *P. unifilis* in Brazil (23, 24). The morphological parameters assessed here showed statistically significant differences between males and females of both species, where females are bigger. These results agree with characteristics described by other authors for *P. vogli* and *P. unifilis* (1, 25).

In both species, the dimension of erythrocytes observed in peripheral blood exceed 24 μm in length and 15 μm in width, making for relatively large cells, only outnumbered by a few species of chelonians, like *Platysternon megacephalum (*25.5 x 15.1 μm)*, Aldabrachelys gigantea (*25 x 15.2 μm)*, Dermochelys coriacea* (24.9 x 15.9 μm), and *Lepidochelys olivacea* (25.7 x 14.4 μm) (26). The size of erythrocytes was similar in both species, which could be a Podocnemidid characteristic; however, there is no report about it in other family members. In this way, it is necessary to carry out studies focused on the cellular morphometry of these species to confirm or refute this hypothesis. Additionally, most of the individuals sampled for both species had basophilic erythrocytic inclusions in the cytoplasm that could be dotted, circular, or ovoid (Figure 1A); these inclusions were previously reported in other species of chelonians and could be remnants of membranes of degenerated organelles, possibly mitochondria's (27).

The leukocytes found in peripheral blood for both species are shown in Figures 1, 2. These leukocytes morphologically agree with different reports for chelonians and other reptiles (22, 28, 29); however, blood cells that coincide with morphological descriptions of azurophils could be observed despite being mainly present in the blood of squamates and crocodilians (22). It is necessary to carry out analyses using other techniques, such as transmission electron microscopy, to determine if these cells correspond to azurophils, as previous photographic reports of this type of cell are quite heterogeneous.

Ninety per cent of the hematological and serum chemistry parameters assessed in *P. vogli* did not show significant statistical differences between males and females; nonetheless, parameters like mean corpuscular volume (MCV), percentage of eosinophils, and concentration of creatinine did show significant differences. Despite this, the difference is only evident when means are compared, as those parameters have a similar range between sexes, except for MCV, which was higher in females (Supplementary Table 1). MCV value is influenced by packed cell volume (PCV) and red blood cell count (RBC); however, significant differences were not observed in these parameters between both sexes in *P. vogli*. Previous studies showed a directly proportional relationship between the carapace length of marine turtles like *Caretta caretta, Chelonia mydas*, and *Eretmochelys imbricata* with hematological parameters such as PCV, erythrocyte size, and MCV (30), which could explain the difference in MCV between sexes.

Similar to *P. vogl*i, 83% of the assessed parameters in *P. unifilis* were alike between males and females, except for the percentage of monocytes and azurophils and the concentrations of AST, cholesterol, and creatinine, which did significantly show differences. The concentration of AST was higher in males, which could be related to the increased activity and aggression shown by male turtles during the mating season (31, 32). On the contrary, females of *P. unifilis* showed a higher cholesterol concentration than males, which could be related to reproductive processes, such as vitellogenesis and egg production (33, 34). For the rest of the parameters with significant differences between sexes in both species, the difference was observed when means were compared since the ranges of distribution of the values were similar between males and females, which may be accentuated by the limited number of individuals sampled of each sex. Besides, when outliers were removed from some parameters of *P. unifilis*, those parameters had fewer than 20 valid observations, whereby, according to the guidelines of the AVSCP, it is not recommendable to establish reference intervals; despite this, we proposed approximate intervals for those parameters anyway, so they should be used cautiously (Table 3).

Most of the hematological and serum chemistry values evaluated were similar between *P. vogli* and *P. unifilis*; however, in *P. unifilis*, less RBC, percentage, and absolute count of eosinophils, as well as low glucose, AST, and cholesterol concentration, were observed, while *P. vogli* showed less MCV and MCH than *P. unifilis*. Considering that erythrocyte size is inversely proportional to RBC (35) and directly proportional to erythrocytic indexes such as MCV (11), these observations support the results shown previously, where *P. vogli*, having smaller erythrocytes, showed higher RBC and lower MCV and MCH, while the opposite was observed in *P. unifilis*. Furthermore, considering that individuals of both species were under the same environmental conditions and that the samples were obtained and processed in the same way and period, the differences observed in parameters like glucose or AST concentration could be caused by the inner characteristics of each species.

Previous hematological studies of *P. unifilis* from Peru showed that RBC and WBC, as well as the percentage of lymphocytes and absolute counts of heterophils, lymphocytes, eosinophils, and azurophils, were higher than those in this present study, while values of the rest of the parameters such as PCV, hemoglobin concentration, and differential count were lower (7, 8). Due to samplings of *P. unifilis* in all studies being made from captive individuals, these differences could be caused by variations in environmental conditions, sampling techniques, and processing.

The RBC showed lower values for *P*. *unifilis* and *P. vogli* than those reported for *Podocnemis expansa, P. unifilis, P. erythrocephala*, and *Peltocephalus dumerilianus* (5–7, 9, 10, 36). On the other hand, for both species studied here, the PCV, TS, hemoglobin concentration, MCV, and MCHC were similar to those reported in *P. expansa* in Brazil (6). Additionally, the glucose, albumin, cholesterol, uric acid, and creatinine concentrations were also similar to those reported for *P. expansa* in Venezuela (5). Unlike these similarities, a marked heterogeneity between the hematological and serum chemistry values was observed for the Podocnemidids family (4, 7, 9, 10, 36), where blood samples were obtained from different venipuncture sites, from organisms belonged to different ages, and were wild or captive turtles. This shows, once again, the difficulties when hematological and serum chemistry parameters of reptiles and other ectothermic animals are assessed and their reference intervals are established, since these animals have different physiologic adaptations in response to intrinsic (species, sex, age, physiological state) and extrinsic (season, temperature, habitat, diet, diseases, stress by captivity, and venipuncture site) factors, hindering their study, analysis, and interpretation (11).

Despite these issues, establishing the baseline for the hematological and blood chemical parameters for endemic or threatened wild or captive species is crucial, as these data could be useful for veterinary diagnostics and for decision-making in conservation programs. Consequently, the hematological intervals proposed here could be considered as a guide for clinic interpretation; nevertheless, values outside of these ranges could not strictly indicate disease; thus, it is necessary to be cautious when using them in veterinary diagnosis.

In conclusion, this study establishes the baseline for the hematological and serum chemistry parameters for *P. vogli* and contributes new data on serum chemistry for *P. unifilis*, which can be used as a reference at the clinical level and for future research, considering that intrinsic and extrinsic factors could alter them. The establishment of these parameters for healthy turtles is crucial for their conservation and management, as this information can be useful for taking decisions and identifying threats, pathogen agents, or environmental factors that could be threatening those species. On the contrary, due to the lifestyles of those turtles in nature, wild individuals of species such as *P. vogli* are commonly infected by blood parasites, which complicates the establishment of reference intervals for these species without falling into biases caused by the infection. Therefore, the development of analysis from healthy captive animals, which are not infected by those microorganisms, could be an effective tool to analyse these parameters and assess the impact of microorganisms on the physiology of turtles.

## Data availability statement

The raw data supporting the conclusions of this article will be made available by the authors, without undue reservation.

## Ethics statement

The animal study was reviewed and approved by the Science Faculty Ethics Committee of the Universidad Nacional de Colombia: N° 03-2019.

## Author contributions

Sampling was carried out by CR-A and GF-R. Sample processing and analyzing, as well as manuscript writing and editing, statistical analyses, graphics, and table assembling, were developed by CR-A. Microscopical analyses, measurements, and diagnostics were developed by LG and GF-R. NM and CM-T did training in methodologies used in field and lab, as well as project schemes. All authors contributed to this study and approved the submitted version.

## Funding

This work was funded by the Research Division of the Universidad Nacional de Colombia under Projects N° 42105 and 50839.

## Conflict of interest

The authors declare that the research was conducted in the absence of any commercial or financial relationships that could be construed as a potential conflict of interest.

## Publisher's note

All claims expressed in this article are solely those of the authors and do not necessarily represent those of their affiliated organizations, or those of the publisher, the editors and the reviewers. Any product that may be evaluated in this article, or claim that may be made by its manufacturer, is not guaranteed or endorsed by the publisher.
