# Supplementary material for: Hematological and blood chemistry parameters of a Podocnemis vogli and P. unifilis captive population in Colombia
Source: Front Vet Sci. 2022 Sep 15;9:961609. doi: 10.3389/fvets.2022.961609 (PMC9521715; doi:10.3389/fvets.2022.961609)
Supplement: Supplementary file 1 [file Table_1.DOCX]

Supplementary Material

# Supplementary Table 1. Comparison of hematological and serum chemistry parameters that showed statistically significant differences between males and females of *Podocnemis vogli* and *P. unifilis*.

| ***Podocnemis vogli*** | | | | | | | | | |
| --- | --- | --- | --- | --- | --- | --- | --- | --- | --- |
| **Parameter** | **Female** | | | | **Male** | | | | ***p-*value** |
|  | **Mean** | **SD** | **Min** | **Max** | **Mean** | **SD** | **Min** | **Max** |  |
| MCV | 1214,1 | 393,3 | 702,3 | 2000,0 | 872,5 | 181,6 | 635,1 | 1222,2 | 0,0119 |
| Eosinophils | 18,8 | 5,8 | 9 | 27 | 13,9 | 5,9 | 5 | 28 | 0,0411 |
| Creatinine | 0,23 | 0,0 | 0,2 | 0,3 | 0,3 | 0,1 | 0,2 | 0,4 | 0,0146 |
| ***Podocnemis unifilis*** | | | | | | | | | |
| **Parameter** | **Female** | | | | **Male** | | | | ***p-*value** |
|  | **Mean** | **SD** | **Min** | **Max** | **Mean** | **SD** | **Min** | **Max** |  |
| Monocytes | 5,3 | 3,2 | 2,0 | 12,0 | 9,5 | 2,3 | 6,0 | 12,0 | 0,01 |
| Azurophils | 0,3 | 0,6 | 0,0 | 0,5 | 1,2 | 0,8 | 0,0 | 1,8 | 0,02 |
| AST | 84,3 | 20,6 | 52,0 | 134,0 | 121,0 | 34,5 | 83,0 | 166,0 | 0,01 |
| Cholesterol | 104,0 | 42,0 | 35,0 | 166,0 | 60,3 | 13,2 | 37,0 | 72,0 | 0,003 |
| Creatinine | 0,3 | 0,1 | 0,1 | 0,4 | 0,2 | 0,1 | 0,2 | 0,4 | 0,008 |
